# The Right Time to Safely Re-Evaluate Empirical Antimicrobial Treatment of Hip or Knee Prosthetic Joint Infections

**DOI:** 10.3390/jcm8122113

**Published:** 2019-12-02

**Authors:** Luc Deroche, Pascale Bémer, Anne-Sophie Valentin, Anne Jolivet-Gougeon, Didier Tandé, Geneviève Héry-Arnaud, Carole Lemarié, Marie Kempf, Laurent Bret, Christophe Burucoa, Stéphane Corvec, Chloé Plouzeau

**Affiliations:** 1 Bacteriology-Hospital Hygiene Department, University Hospital of Poitiers, Poitiers Universiy, F-86000 Poitiers, France; Christophe.burucoa@chu-poitiers.fr (C.B.); Chloe.PLOUZEAU-JAYLE@chu-poitiers.fr (C.P.); 2 Bacteriology-Hospital Hygiene Department, University Hospital of Nantes, Nantes University, F-44000 Nantes, France; pascale.bemer@chu-nantes.fr (P.B.); stephane.corvec@chu-nantes.fr (S.C.); 3 Bacteriology-Hospital Hygiene Department, University Hospital of Tours, Tours University, F-37000 Tours, France; as.valentin@chu-tours.fr; 4 Univ Rennes, INSERM, University Hospital of Rennes, NUMECAN Institute (Nutrition Metabolisms and Cancer), F-35000 Rennes, France; anne.gougeon@chu-rennes.fr; 5 Bacteriology-Hospital Hygiene Department, University Hospital of Brest, Brest University, F-29000 Brest, France; didier.tande@chu-brest.fr (D.T.); Genevieve.Hery-Arnaud@univ-brest.fr (G.H.-A.); 6 Bacteriology-Hospital Hygiene Department, University Hospital of Angers, Angers University, F-49000 Angers, France; CaLemarie@chu-angers.fr (C.L.); makempf@chu-angers.fr (M.K.); 7 Bacteriology-Hospital Hygiene Department, Hospital of Orléans, F-45000 Orléans, France; laurent.bret@chr-orleans.fr

**Keywords:** prosthetic joint infection, bacterial growth time, time to positivity, empirical antimicrobial treatment, postoperative antibiotics

## Abstract

Currently, no guideline provides recommendations on the duration of empirical antimicrobial treatment (EAT) in prosthetic joint infection (PJI). The aim of our study was to describe the time to growth of bacteria involved in PJI, rendering possible decreased duration of EAT. Based on a French multicentre prospective cohort study, culture data from patients with confirmed hip or knee PJI were analysed. For each patient, five samples were processed. Time to positivity was defined as the first positive medium in at least one sample for virulent pathogens and as the first positive medium in at least two samples for commensals. Definitive diagnosis of polymicrobial infections was considered the day the last bacteria were identified. Among the 183 PJIs, including 28 polymicrobial infections, microbiological diagnosis was carried out between Day 1 (D1) and D5 for 96.7% of cases. There was no difference in the average time to positivity between acute and chronic PJI (*p* = 0.8871). Microbiological diagnosis was given earlier for monomicrobial than for polymicrobial infections (*p* = 0.0034). When an optimized culture of peroperative samples was carried out, almost all cases of PJI were diagnosed within five days, including polymicrobial infections. EAT can be re-evaluated at D5 according to microbiological documentation.

## 1. Introduction

The management of prosthetic joint infection (PJI) is a major challenge for physicians, as its consequences can be devastating for the patient. One of the key points in the treatment strategy is time of microbiological diagnosis. Pre-operative samples may be useful for identifying bacteria before surgery, but documentation may be incomplete, compared to peroperative samples [1]. An empirical antimicrobial treatment (EAT) with antibiotics is settled after surgery in order to cover the bacteria most frequently isolated from PJI. A combination containing a beta-lactam, such as piperacillin/tazobactam or a third-generation cephalosporin, is recommended, associated to an antibiotic that has been shown to be effective against Gram-positive bacteria, methicillin-resistant staphylococci in particular [2,3,4,5]. Vancomycin, daptomycin or linezolid are suitable options for this application [6,7,8]. Bacterial identification tools allow a faster diagnosis, but no recommendation provides guidelines on the duration of this EAT and the right time to re-evaluate it [2,3,9]. The probabilistic antibiotics often used are not harmless, and severe adverse effects can occur, such as nephrotoxicity when the vancomycin being used is associated with piperacillin/tazobactam [10,11]. The goal of the present study was to describe the growth time of bacteria involved in hip or knee PJI in order to find out when EAT can be safely re-evaluated, according to microbiological results.

## 2. Materials and Methods

### 2.1. Study Population

From December 2010 to March 2012, 204 consecutive patients from seven French medical centres with confirmed hip or knee PJI were enrolled in a multicenter, prospective, observational, cross-sectional study [12]. All patients had five surgical samples. The present study is a secondary analysis of these data, focusing on 183 patients with culture-positive PJI. 

### 2.2. Definition of PJI

Early postoperative PJI was suspected for patients with pain, disunion, necrosis, or wound dehiscence within the first month following prosthesis implantation. Late chronic PJI was suspected in the presence of chronic pain, as well as a loosened prosthesis, occurring more than 1 month after the index surgery [13,14]. In accordance with the Infectious Diseases Society of America (IDSA) guidelines, PJI was diagnosed when at least one of the following criteria was positive: (i) clinical criterion with the presence of a sinus tract communicating with the prosthesis and/or purulence around the prosthesis, and/or (ii) bacteriological criterion for infection (as specified below). 

In the present study, only microbiologically documented PJIs were analysed.

### 2.3. Laboratory Analysis

Each solid sample was crushed by stainless-steel beads, on a Retsch MM401 (Retsch Technology Gmbh, Haan, Germany), after the addition of 10 mL of sterile water. One paediatric blood culture and a thioglycolate liquid broth (Schaedler) were inoculated with 1 mL and incubated for 14 days. A blood agar plate and a PolyViteX chocolate agar plate were inoculated with 50 µL and incubated for 7 days under a CO_2_-enriched atmosphere. Another blood agar plate was inoculated and incubated for 7 days in an anaerobic atmosphere. Joint fluids were inoculated on the same media [12].

Time to positivity was defined as the time of growth of the first positive medium in at least one sample for pathogens such as *Staphylococcus aureus*, *Pseudomonas aeruginosa*, *Enterobacterales*, or anaerobes (excluding *Cutibacterium acnes*), and the time of growth of the first positive medium in at least two samples for commensals such as coagulase-negative staphylococci (CoNS) or *C. acnes*. The sustained diagnosis day of polymicrobial infections was the time of growth of the last identified bacteria.

### 2.4. Statistical Analysis

Pearson *Χ*^2^ test or Fisher’s exact test were performed to compare qualitative variables, according to sample sizes. A mean comparison of time of growth was performed with an unpaired Student’s *t* test. A *p*-value < 0.05 was considered statistically significant.

### 2.5. Ethics

The study protocol (PHRCI API/N/041) was approved by the institutional review board and ethics committee. Informed consent was obtained from each patient before inclusion.

## 3. Results

Among the 183 confirmed PJIs analysed, 28 were polymicrobial. Patient characteristics are detailed in Table 1. No significant difference was observed between the clinical features of monomicrobial and polymicrobial infection groups.

Time to growth was analysed for each medium of the five samples of each patient, representing 4575 culture media. Detailed data of time to positivity for each bacterium is available in Table 2. All isolates of *S. aureus*, streptococci, enterococci and *Pseudomonas aeruginosa* were retrieved by culture in five days or less. Among other bacterial species, only six bacteria grew between D5 and D14: two *C. acnes*, one *Enterobacter cloacae*, one *S. epidermidis*, one *Corynebacterium urealyticum* and one *Prevotella* sp. Three out of six patients concerned by late culture (two at D7 and one at D14) had received antibiotics in the previous 15 days. One of the *C. acnes* infections diagnosed at D7 already had one positive sample at D4.

When considering each bacterial species separately, time of growth was similar in both monomicrobial and polymicrobial infections. Also, there was no difference in mean time to positivity between acute and chronic infections (mean of 1.545 and 1.586, respectively; *p* = 0.8871).

Table 3 represents the day of microbiological diagnosis, for each patient (every culture media from every sample of the same patient was included), including polymicrobial data. Microbiological diagnosis was given between D1 and D5 for 96.7% (177/183) of cases (monomicrobial: 97.4% (151/155); polymicrobial: 92.9% (26/28)). Definitive diagnosis was given earlier in monomicrobial than in polymicrobial infections (*p* = 0.0034).

## 4. Discussion

The present work is the first multicentre study focused on time to positivity of PJI samples. The main finding is that 96.7% of PJIs with positive culture were diagnosed between day 1 (D1) and D5, including polymicrobial infections, when cultures are optimized (beadmill processing, inoculation of blood culture).

There is strong evidence that when introduced after surgery, EAT is associated with a higher remission rate [15,16]. This empirical treatment should ensure wide coverage of the bacteria responsible for PJI. While current guidelines do not provide any advice on the duration of empirical treatment, they do recommend bacterial cultivation time up to 14 days [2,3]. Keeping the cultures two weeks is sufficient to recover almost all of the bacteria conventionally involved in PJI, including anaerobes [12,17]. The isolation of *C. acnes* remains a difficult topic in implant-associated infections, but it seems that in proven PJI due to this bacteria, cultures are positive in 5 to 7 days, whereas they are positive later in cases of plate contamination [18,19].

Infection can be documented before surgery, by aspiration of synovial fluid or collection of blood cultures. The specificity of pre-operative aspiration culture for infection diagnosis is very high, but its sensitivity remains moderate [1]. Moreover, the identified bacterial species may not reflect the exact microbiology of the infection [20]. As a result, it is unwise to narrow the spectrum of EAT to pre-operative samples.

In this study, the definitive bacterial diagnosis was given earlier for monomicrobial than for polymicrobial infections, but almost all infections were diagnosed between D1 and D5. Taking these results into account, when cultures are optimized (beadmill processing, inoculation of blood culture), EAT can be switched to an adapted treatment as soon as the fifth day of laboratory culture.

Our results may not be transposable to PJI infections other than hip and knee. As the proportion of skin commensals like *C. acnes* is higher in shoulder PJI, further studies are needed to describe the time of growth of bacteria found in other PJI locations [19,21].

**Figure 1:**
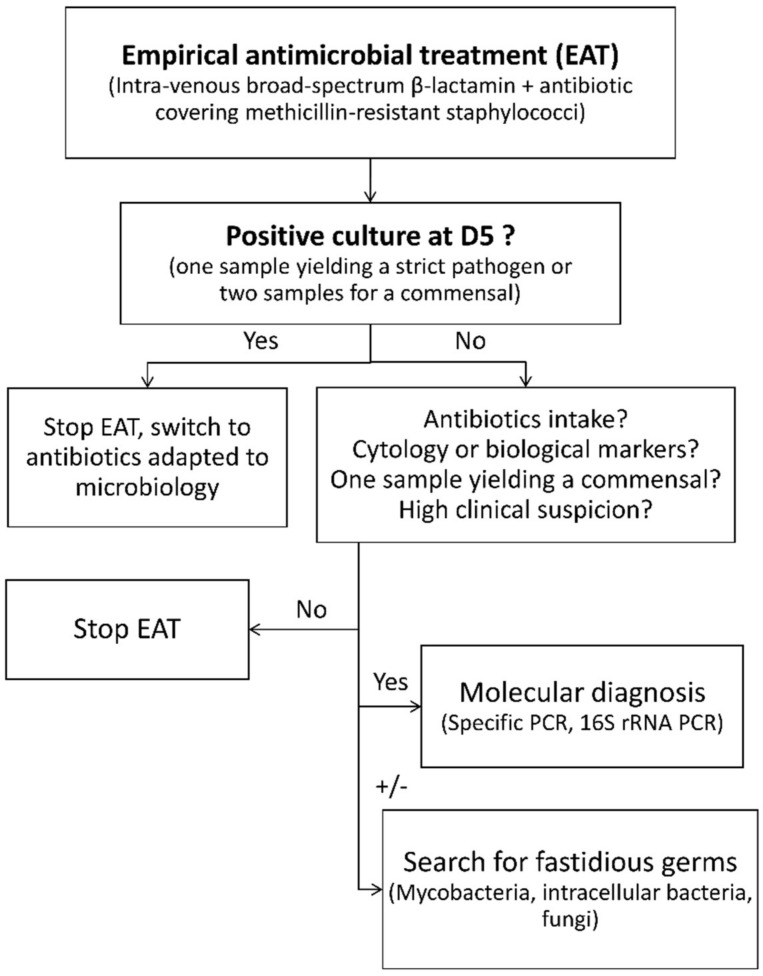
Algorithm proposal for the time of re-evaluation of empirical antimicrobial treatment and supplementary microbiological diagnosis of hip or knee prosthetic joint infection.

Two other teams have suggested new approaches designed to adapt probabilistic treatment. Benito and colleagues focused on the influence of route of acquisition and time after surgery on the microbiology of PJI [22]. In their prospective study, Triffault-Fillit and colleagues described the microbiology of PJI depending on time to occurrence [23]. These two studies suggest that few or no Gram-negative bacilli are isolated from late PJI. We think these data are complementary to our results. In our study, no conclusion can be made on hematogenous acute infection, as the relevant clinical data were not available. However, as no difference was observed between acute or chronic infection, we think our results can be applied to every situation of hip or knee PJI, regardless of the EAT chosen. The 7-day threshold may seem safer, but only four bacteria grew between D5 and D7 (three monomicrobial infections, one polymicrobial). Among them, two out of four (50%) patients received antibiotics in the previous 15 days, and one *C. acnes* grew in one sample at D4. In the case of negative cultures, modification or discontinuation of EAT should be discussed, as described in the decision algorithm suggested in Figure 1. 

This algorithm can be used in any centre already performing optimized bacterial culture for PJI diagnosis, as it enables faster bacterial growth and higher sensitivity and specificity [12,24,25]. When following this algorithm, four out of six infections diagnosed after D5 can be anticipated (three had antibiotics, one had a positive sample at D4), without taking into account other factors such as biological markers (not available in this study). When cultures are negative at D5, molecular testing (bacteria-specific PCR, 16S rRNA PCR) should be considered earlier, in the case of antibiotic intake, high clinical suspicion of PJI, presence of a minor criteria from the latest Musculoskeletal Infection Society (MSIS) definition or if histopathology is in favour [12,26,27]. These criteria include both serum (C-reactive protein (CRP), D-dimer and erythrocyte sedimentation rate) and synovial markers (white blood cells count, polymorphonuclear percentage, leucocyte esterase, alpha-defensin and CRP). The search for fastidious germs, such as moulds or mycobacteria, may be discussed, as it requires specific media [28]. Intracellular bacteria, like *Mycoplasma*, are rare causes of PJI, but should be considered and searched with a specific PCR [29]. This strategy may save time, as specific research can be considered earlier. 

Oral antibiotics may be used when re-evaluating EAT, as the Oral versus Intravenous Antibiotics for Bone and Joint Infection (OVIVA) trial demonstrated that switching to an oral regiment within 7 days after surgery was noninferior to intravenous antibiotic treatment, with significant cost savings [30,31]. Lack of stable venous access is a common difficulty that justifies earlier adaptation of the EAT. In addition to the benefits of early oral antibiotic administration, a shorter EAT should reduce the number of adverse effects due to broad-spectrum antibiotics. The combination of piperacillin/tazobactam plus vancomycin is becoming more and more widely criticized due to the increased risk of kidney injury compared to other broad-spectrum beta-lactams associated with vancomycin [10,11,32,33]. However, a short course does not seem to be associated with an increased risk of adverse renal outcomes [34]. A shorter probabilistic treatment may also be beneficial in the case of linezolid use, as it limits the development of drug resistance and reduces side-effects, which are associated with the treatment duration [7,8,35,36,37,38].

## 5. Conclusions

Our results suggest that the EATs of hip or knee PJI can be adapted at the fifth day of an optimized culture (beadmill processing, inoculation of blood culture). This will reduce the duration of the EAT used in PJI.

## Figures and Tables

**Table 1:** Clinical features of the 183 cases of hip or knee prosthetic joint infection, according to the monomicrobial or polymicrobial type of infection.

Variable	Monomicrobial Infections (*n* = 155)	Polymicrobial Infections (*n* = 28)	All Infections (*n* = 183)
Mean age (year +/- SD)	71.4 ± 13.2	70.3 ± 11.6	71.5 ± 13.3
Male sex (no. [%])	85 (54.8)	12 (42.9)	97 (53.0)
Location of arthroplasty (no. [%])			
Knee	53 (34.2)	6 (21.4)	59 (32.2)
Hip	102 (65.8)	22 (78.6)	124 (67.8)
Presentation of infection (no. [%])			
Acute infection (<1 month)	33 (21.3)	10 (35.7)	43 (23.5)
Chronic infection (>1 month)	122 (78.7)	18 (64.3)	140 (76.5)
Type of surgery (no. [%])			
Debridement with retention	48 (31.0)	11 (39.3)	59 (32.2)
One-stage technique	43 (27.7)	4 (14.3)	47 (25.7)
Two-stage technique	52 (33.5)	9 (32.1)	61 (33.3)
Permanent explantation of joint prosthesis	5 (3.2)	3 (10.7)	8 (4.4)
Data not available	7 (4.5)	1 (3.6)	8 (4.4)
Number of previous joint surgeries (no. [%])			
1	83 (53.5)	16 (57.1)	99 (54.1)
2	49 (31.6)	5 (17.9)	54 (29.5)
≥3	16 (10.3)	6 (21.4)	22 (12.0)
Data not available	7 (4.5)	1 (3.6)	8 (4.4)
Antibiotherapy in the 15 days before surgery (no. [%]) ^a^	44 (28.4)	10 (35.7)	54 (29.5)
β-lactams	21 (47.7)	5 (50.0)	26 (48.1)
Pristinamycin	3 (6.8)	0	3 (5.6)
Clindamycin	2 (4.5)	0	2 (3.7)
Rifampin	4 (9.1)	0	4 (7.4)
Fluoroquinolones	8 (18.2)	1 (10.0)	9 (16.7)
Cotrimoxazole	5 (11.4)	2 (20.0)	7 (13.0)
Others	13 (29.5)	3 (30.0)	16 (29.6)

^a^, some patients received two antibiotics; No significant difference in clinical features was observed between monomicrobial and polymicrobial infections; Percentages may not total 100 because of rounding.

**Table 2:** Time of growth for each type of bacteria isolated in hip and knee PJI, according to the monomicrobial (M) or polymicrobial (P) type of infection.

	*S. aureus* (*n* = 75)		CoNS * (*n* = 58)		Streptococci (*n* = 32)		Gram-Negative Bacilli (*n* = 32)				Anaerobes (*n* = 8)		*C. acnes* (*n* = 7)		Others ** (*n* = 11)	
							*Enterobacterales* (*n* = 26)		*P. aeruginosa* (*n* = 6)							
Mono- or Polymicrobial infection	M	P	M	P	M	P	M	P	M	P	M	P	M	P	M	P
Day 1	54	12	38	10	19	10	10	14	2	2	1				4	3
Day 2	2	1	6	3	3		1		2		1	4				2
Day 3		1											2			
Day 4													1			1
Day 5	2	1										1	1	1		
Day 7			1				1					1	1			
Day 14													1			1
	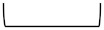		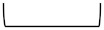		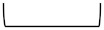		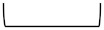									
*p*-value	0.2135		0.8946		0.2332		0.2176		NC		NC		NC		NC	

*, CoNS: coagulase-negative staphylococci; **, Others: *Corynebacterium sp.* (*n* = 6), *Listeria monocytogenes* (*n* = 2), *Bacillus cereus* (*n* = 1), *Actinomyces* sp. (*n* = 1), *Capnocytophaga canimorsus* (*n* = 1); M: Monomicrobial infections; P: Polymicrobial infections; *p*-values were used to compare the mean time of growth in monomicrobial versus polymicrobial infections; NC: not calculated due to low *n*.

**Table 3:** Cumulative number of infections diagnosed, according to the day of bacterial culture (monomicrobial or polymicrobial prosthetic joint infection).

	Monomicrobial (*n* = 155)		Polymicrobial (*n* = 28)		Both (*n* = 183)	
	*n*	%	*n*	%	*n*	%
Day 1	130	83.9	16	57.1	146	79.8
Day 2	146	94.2	21	75.0	167	91.3
Day 3	147	94.8	22	78.6	169	92.3
Day 4	148	95.5	23	82.1	171	93.4
Day 5	151	97.4	26	92.9	177	96.7
Day 7	154	99.4	27	96.4	181	98.9
Day 14	155	100.0	28	100.0	183	100.0
